# Does Reproductive Investment Decrease Telomere Length in *Menidia menidia*?

**DOI:** 10.1371/journal.pone.0125674

**Published:** 2015-05-04

**Authors:** Jin Gao, Stephan B. Munch

**Affiliations:** 1 Department of Ecology and Evolution, Stony Brook University, Stony Brook, NY, United States of America; 2 School of Marine and Atmospheric Sciences, Stony Brook University, Stony Brook, NY, United States of America; CNRS, FRANCE

## Abstract

Given finite resources, intense investment in one life history trait is expected to reduce investment in others. Although telomere length appears to be strongly tied to age in many taxa, telomere maintenance requires energy. We therefore hypothesize that telomere maintenance may trade off against other life history characters. We used natural variation in laboratory populations of Atlantic silversides (*Menidia menidia*) to study the relationship between growth, fecundity, life expectancy, and relative telomere length. In keeping with several other studies on fishes, we found no clear dependence of telomere length on age. However, we did find that more fecund fish tended to have both reduced life expectancy and shorter telomeres. This result is consistent with the hypothesis that there is a trade-off between telomere maintenance and reproductive output.

## Introduction

Telomeres are regions of DNA at the end of a chromosome that facilitate chromosome replication. Telomere shortening limits somatic cells to a finite number of cell divisions [1, 2]. Since regular polymerases are unable to extend the ends of chromosomes [3], maintaining telomere length requires telomerase activity. Both telomerase and shortening processes are normally tightly regulated [4] such that telomere length declines consistently with age in many species.

In birds, telomere length decreases with age in black-backed gulls (*Larus fuscus*, [5]), corvids (*Corvus monedula*, [6]), and zebra finch (*Taeniopygia guttata*, [7]). This is sufficiently consistent that telomere length can be used as a predictor of age in chickens [8]. Consistent telomere shortening is not restricted to homeotherms. Telomere length declines with body size and age in alligators [9] as well as in California garter snakes (*Thamnophis elegans* [10]. Hatase [11] studied the telomere dynamics in loggerhead turtle (*Caretta caretta*) and found that telomere length vs. age relationship is tissue dependent; telomeres decrease with size and age in epidermal tissues, but do not shorten with age in blood.

In fishes, however, the age-dependence of telomere length is far less clear [12–24]. The majority of fish telomeres studied have been in freshwater species, primarily zebrafish (*Dano rerio*, [12–14]), Japanese medaka (*Oryzias latipes*, [15, 16]), African killifish (*Nothobranchis furzeri*, [17]), platyfish (*Xiphophorus*, [18]) and eastern mosquito fish (*Gambusia holbrooki*, [19]) and the results for fishes are apparently more complicated than those for other taxa. For instance, in zebrafish telomere length did not decline steadily over time [12] but then drastically declined in aged fish [13] while the telomerase activity remained high throughout the entire life [12, 13]. In African killifish, strains with longer lifespan exhibit age-dependent telomere shortening while short-lived strains do not [17]. In eastern mosquito fish, telomere seems to shorten with age in lab populations [19]. Sex differences in telomere length have been identified in medaka [20] and common carp (*Cyprinus carpio*) [21]

To our knowledge, telomere length has only been studied in three marine fishes. Atlantic cod (*Gadus morhua*) caught in the North Sea showed gender-specific differences in telomere length in liver samples [22]. European hake (*Merluccius merluccius*) showed great variability in telomere length in the brain, muscle, kidney and liver at the individual level and thus alone cannot be used as a reliable biomarker for age [23]. Horn et al. [24] studied the European sea bass (*Dicentrarchus labrax*) from 12 to 92 months old in the lab and did not find any evidence for telomere shortening. There was, however, substantial variation in telomere length among individuals.

Assuming that the dynamics of telomere length and their consequences for cell replication are similar across birds, reptiles, and fishes, we should ask why telomeres fail to show a consistent decline with age in fishes. Reznick et al. [25] argued that senescence should be delayed if fecundity increases sufficiently rapidly with age. Prolonged telomere maintenance would be a necessary corollary of such a strategy. Moreover, it suggests that rates of telomere shortening may be adaptive and may trade-off against other energetically costly components of fitness [24, 26]

Using Atlantic silversides as a model system, we tested whether telomere length shortens with age and whether there are apparent trade-offs between growth, fecundity, and relative telomere length. Atlantic silversides, *Menidia menidia*, are marine fish common in North American estuaries from Newfoundland to northeast Florida [27]. Breeding occurs in the spring, during which silversides spawn multiple times [28]. Juveniles achieve approximately 90% of their lifetime growth prior to their first winter [29]. Ninety percent of fish mature at age one, and relatively few survive to a second year [30].

## Methods

To determine whether telomere length decreases with age in Atlantic silversides, we grew wild individuals under constant laboratory conditions over 200 days. We used naturally occurring covariation in growth, reproductive investment, and telomere length to evaluate potential trade-offs among these traits.

Eggs were collected from intertidal root masses in the Annapolis Royal Basin (collection permit issued by Fisheries and Oceans Canada) in 2009 (N 44°48’714”, W 65°21’582”) and brought back to Flax Pond Marine Lab, Old Field, New York. These eggs were hatched in the laboratory. Larvae were all collected on the same date and reared in 18L containers from hatch to 60 days during which they were fed to satiation daily using a combination of dry food (Otohime larval feeds, Reed Mariculture) and freshly hatched *Artemia nauplii* (Brine Shrimp Direct). At 60 days, the fish were transferred to 3785L round polyethylene tanks. At 150 days the fish were split haphazardly into four replicates of 22 females and 20 males in each tank. The initial lengths of fish in the four tanks are shown in Table 1. Juvenile and adult fish were fed a mixture of dry food (Otohime larval feeds, Reed Mariculture) and frozen brine shrimp (Brine Shrimp Direct). From day 150 to 200, the total number of eggs produced by the females in each tank was counted on a weekly basis. Fish were maintained at 21(+/- 1.2) ^o^C throughout their lives. These procedures above were carried out under an established protocol approved by the Institutional Animal Care and Use Committee (IACUC) at Stony Brook University.

**Table 1:** The initial lengths (mm) of fish in the four replicates.

	Female		Male	
Tank 1	85.22	+/-10.52	85.12	+/-6.46
Tank 2	88.88	+/-7.02	86.94	+/-6.17
Tank 3	89.26	+/-7.46	85.53	+/-11.27
Tank4	88.61	+/-6.10	86.71	+/-7.45

Both the means and the standard deviations are included.

Fish were subsampled on days 1, 150, and 200 to track the dynamics of telomere length in the population (Table 2). DNA was extracted using a Wizard Genomic DNA Purification Kit (Promega). Because day old larvae were too small to analyze individually, genomic DNA was extracted from 13 pooled samples of 20 larvae each. For juveniles (day 150, n = 5 per gender) and adults (day 200, n = 7 per tank), telomeres were extracted from muscle and brain tissue from individual fish. The 7 fish were a random sample from the remaining (7, 10, 8 and 26 for tank 1–4) fish at day 200 and may not represent the real sex ratio in each tank.

**Table 2:** The sampling plan and number of samples collected.

	Muscle	Brain	Whole organisms	Length	Gonad weight
Day 1	-	-	13 (pooled)	-	-
Day 150	10	10	-	10	10
Day 200	28	28	-	28	28

Muscle and brain tissues could not be analyzed separately for larval fish, nor could total. Different tissues were sampled at different time points.—indicates that the sample is not available.

To evaluate trade-offs between life history characters, gonads for each fish sampled on day 200 were dissected out and dried at 60°C for 48 hours before weighing. Dry gonad weight was used to calculate the gonosomatic index (GSI = dry gonad mass/total dry mass). To estimate average growth over the duration of the experiment, the standard length (+/-0.05mm SD) for each individual was measured using digital calipers.

### Telomere length assay

Real-time polymerase chain reaction was used to measure the overall abundance of telomere repeats as described by Cawthon [31]. Real time quantitative PCR determines, the C_t_ for each sample well: the cycle number at which the well’s accumulating fluorescence crosses a set threshold that is several standard deviations above baseline fluorescence [32]. The linear relationship between C_t_ versus ln[amount of input target DNA] allows the relative quantitation by comparison the C_t_ to a standard curve based on the amplification of a single copy gene.

Telomere (T) PCRs and single copy gene (S) PCRs were performed in separate transparent 96-well plates (Fisher Scientific). Repeated measures of the T/S ratio in the same DNA sample gave the lowest variability when the sample well position for T on the first plate matched the well position for S on the second plate. Two ‘master-mixes’ (SYBR GreenER qPCR SuperMix Universal, Invitrogen) were created for S and T respectively. Detailed protocol and method validation is in **S1 Complete Experimental Protocol**. The well arrangement of T and S PCRs were identical except for the oligonucleotide primers. The T primer sets used were tel1: GGTTTTTGAGGGTGAGGGTGAGGGTGAGGGTGAGGGT and tel2: TCCCGACTATCCCTATCCCTATCCCTATCCCTATCCCTA. The single copy gene used was melanocortin type 1 receptor (Mc1r). This gene was cloned and sequenced in *M*. *menidia*. Although we did not determine copy number for Mc1r in *Menidia*, it is known to be a single copy gene in many teleost species [33]. The primer set for Mc1R (150bp) is forward: GTCCTCCCTCTCGTTCCTGT and reverse: AAGAGGATGCTGGACGTGAT.

All PCRs were performed on Mastercycler ep realplex 2 S system (Eppendorf), a thermal cycler equipped to excite and read emissions from fluorescent molecules during each cycle of the PCR. The thermal cycling profile for both amplicons began with a 95°C incubation for 10 min to activate the AmpliTaq Gold DNA polymerase. For telomere PCR, there followed 40 cycles of 95°C for 15 s and 54°C for 2 min. Similarly for S, optimal conditions were 40 cycles of 95°C for 15 s and 56°C for 30s. Eppendorf’s Realplex 2.0 generated the standard curve for each gene. The T/S ratio was determined based on the Ct value for each sample.

### Trade-off between reproduction and lifespan

To follow up on the idea that energetic allocation to reproduction reduces survival in silversides, we tracked the survival of 86 mature, wild-caught silversides collected from New York (collection permit issued by the New York State Department of Environmental Conservation) June 2008 and held in the lab at 21°C on *ad libitum* rations. Length, sex, and GSI were recorded for each fish on the day of death.

### Statistical analyses

All of the statistical analyses were carried out in Matlab 2012a (Mathworks). To test whether telomeres shorten with age, linear regression was performed on the RTLs collected on days 1, 150, and 200. This was done separately for brain and muscle samples, using the pooled RTL for larvae as the estimate on day 1 for both. To further test for tissue-specific and gender-specific differences in RTL, a paired t-test was performed on all on the samples collected at day 150 and 200.

To test for trade-offs between growth, reproductive investment, and telomere maintenance, we tested for significant regression of telomere length on size and total gonad weight. The slopes and associated standard error were reported in the Results section. Since all fish in the experiment hatched on the same date, final size is an index of the average growth rate over 150 days. To see that this must be true, note that Cov(growth, final size) = [Var(final size)-Cov(initial size, final size)]/time so that the correlation between growth and final size is Corr(growth, final size) = [1-rS]/[1-2rS+S^2^]^1/2^ where r is the correlation between initial and final size and S = SD(initial size)/SD(final size). Although we don’t know r, a little calculus shows that the correlation between growth and final size cant be any smaller than (1-S^2^)^1/2^ = 0.92.

To follow up on the apparent trade-off between telomere length and gonad weight, we evaluated whether differences in net egg production were correlated with RTL. We regressed the average RTL for each tank against the total number of eggs collected from days 150–200. The relationship between mean length and fecundity were also tested via linear regression. To assess the cost of reproductive investment in the trade-off study, we regressed log GSI on the time to death.

## Results

**Fig 1:**
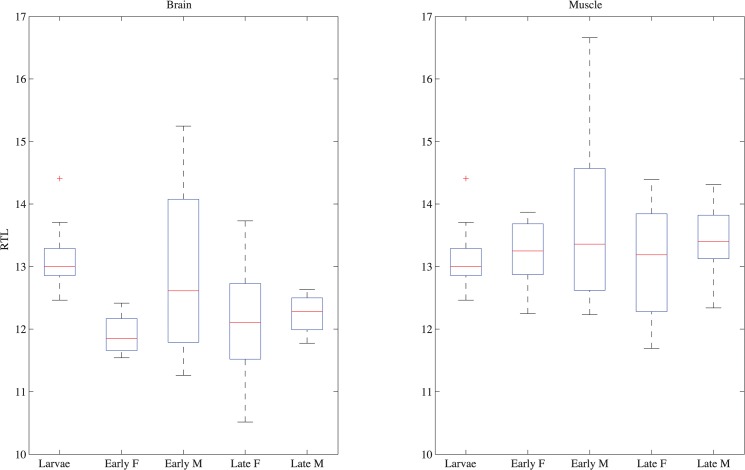
Telomere length through time. The relative telomere length is reported for larvae (age 1 D), females and males from the early breeding period, age 150 days (Early F and Early M respectively), and females and males from late breeding period, 200 days (Late F and Late M respectively). The left panel indicates results for brain tissue and the right panel indicates telomere lengths for muscle. The red line for each box indicates the mean, box edges indicate 25% and 75% percentile, and the whiskers show outlier outside +/-2.7 standard deviation.

**Fig 2:**
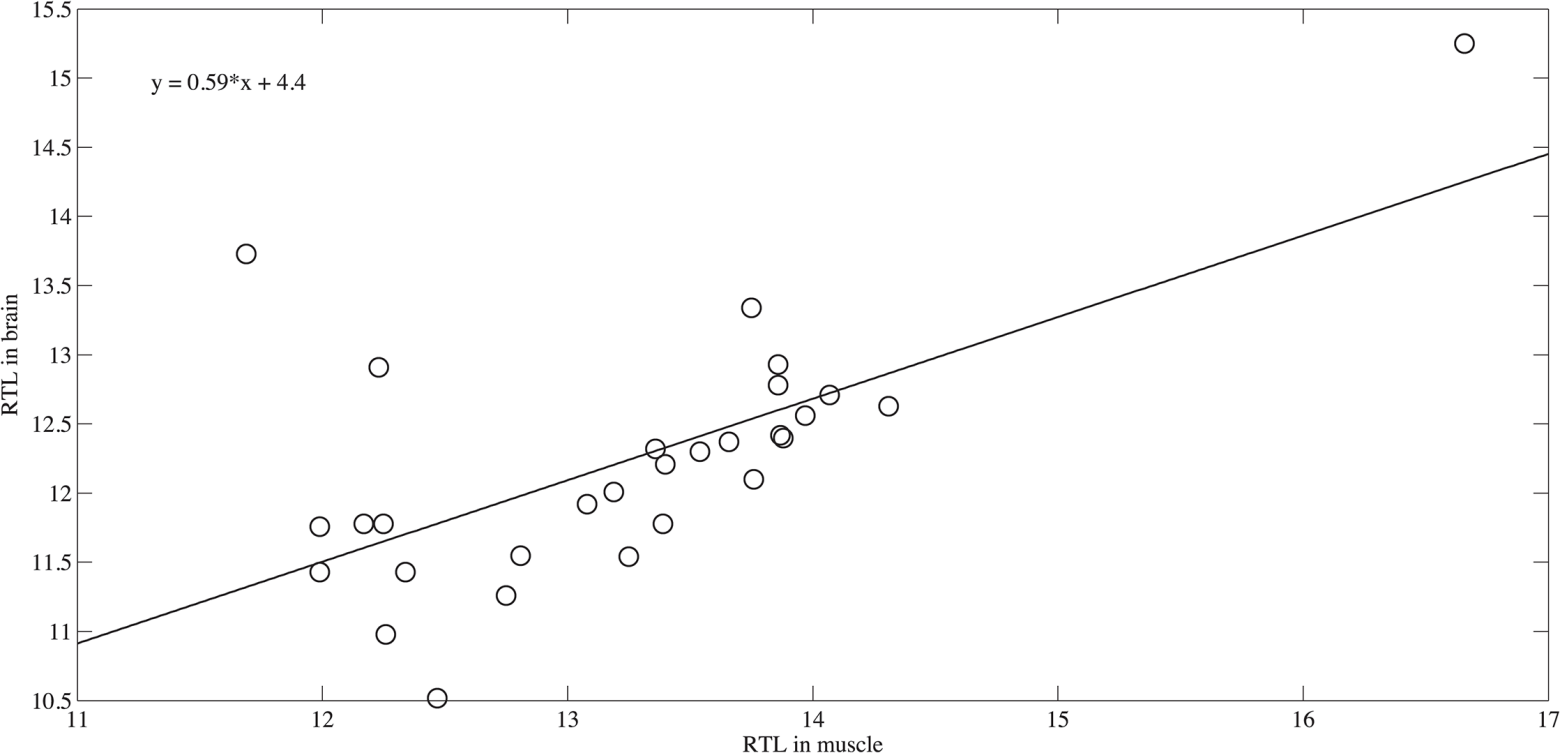
Correlation in RTL between brain and muscle tissue samples. Although differences between tissues were tested using a paired t-test, a linear regression is shown here for visualization.

In keeping with results for other fishes, there was no clear age-dependent trend in the telomere length (Fig 1) in either brain (♀ slope = -0.0060 +/-0.003 R^2^ = 0.83, p = 0.61 and ♂ slope = -0.0040+/-0.003 R^2^ = 0.65, p = 0.45) or muscle (♀slope = 0.000010+/-0.0001 R^2^ = 0.0040, p = 0.73 and ♂ slope = 0.0020+/-0.02 R^2^ = 0.50, p = 0.68) tissue. Moreover, no significant differences were found between the males and females at a given age (brain t = 0.72, p = 0.76, muscle t = -0.31, p = 0.38). Although telomeres in muscle were significantly longer than in brain tissue (t = 4.0, p<0.001), they were highly correlated (slope = 0.59+/-0.13, R^2^ = 0.42, p<0.001, Fig 2). Thus the ample variation among individuals seen in Fig 1 likely represents consistent individual differences in initial telomere length and telomere maintenance rather than measurement noise.

**Fig 3:**
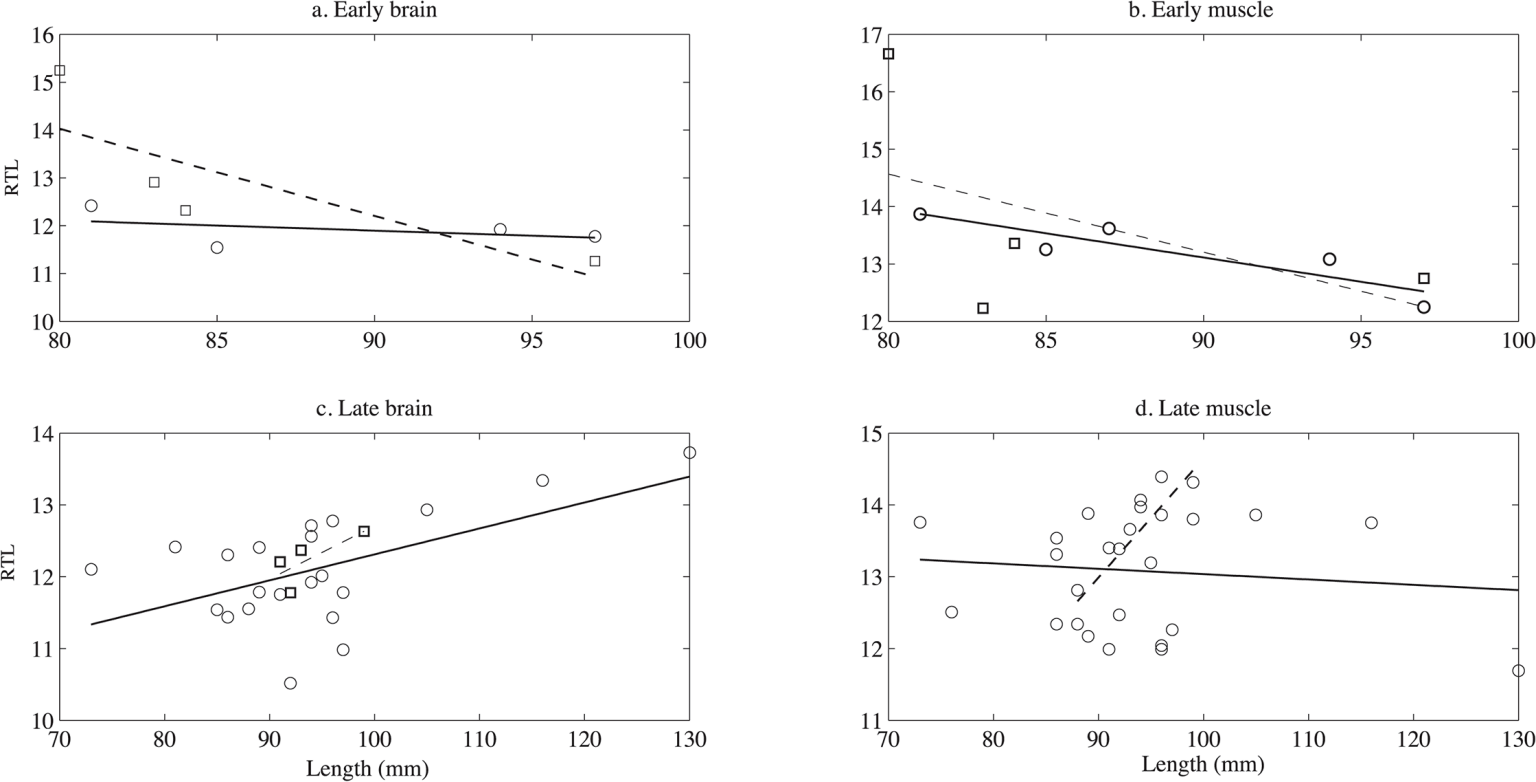
Relationship between RTL and growth history (final length). The RTL from both brain and muscle tissue from fish at the early breeding period is shown in a and b. The RTL from both brain and muscle tissue from fish at the late breeding period is shown in c and d. The dashed lines and solid lines are fitted linear regressions for female samples and male samples respectively.

To test for trade-offs between growth, reproductive investment, and telomere maintenance, we regressed RTL on final length and dry gonad mass. There was no consistent significant pattern to the relationship between body size and telomere length (Fig 3, see Table 3 for standard error and R^2^). In females from day 150, telomeres in muscle, but not brain, decreased with length (muscle: slope = -0.084, p = 0.04, brain: slope = -0.021, p = 0.57). Conversely, in females from day 200 telomeres in brain, but not muscle, increased with length (muscle: slope = -0.0070, p = 0.64, brain: slope = 0.036, p = 0.02). Among males, muscle telomeres increased with size on day 200 (slope = 0.17, 0 = 0.01) but had no other significant dependence on length.

**Table 3:** The regression statistics of RTL on final length and dry gonad mass separated by tissue and gender.

		Early Muscle	Early Brain	Late Muscle	Late Brain
Female	slope	**-0.08**	-0.02	-0.01	**0.04**
	SE	0.02	0.03	0.02	0.01
	R^2^	0.80	0.19	0.01	0.36
	p	0.04	0.57	0.64	0.01
Male	slope	-0.14	-0.18	**0.17**	0.07
	SE	0.16	0.09	0.03	0.05
	R^2^	0.26	0.66	0.89	0.55
	p	0.49	0.19	0.02	0.26

The significant values are in bold and SE stands for standard error.

**Fig 4:**
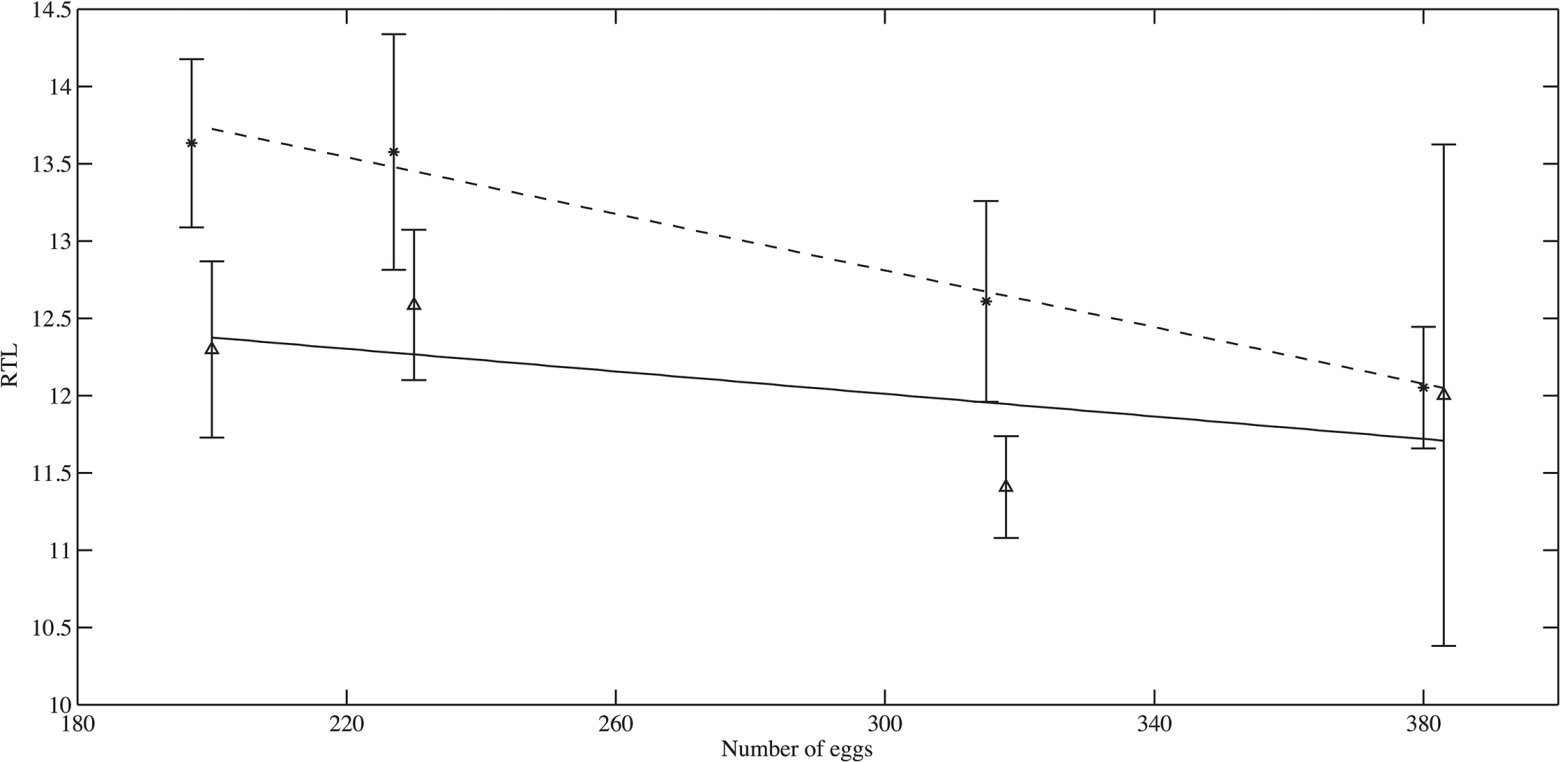
Correlation between average female fecundity and average telomere length across tanks. Muscle samples are marked with star and brain samples are marked with triangle. The dashed line and solid line is linear fit to muscle samples and brain samples respectively.

**Fig 5:**
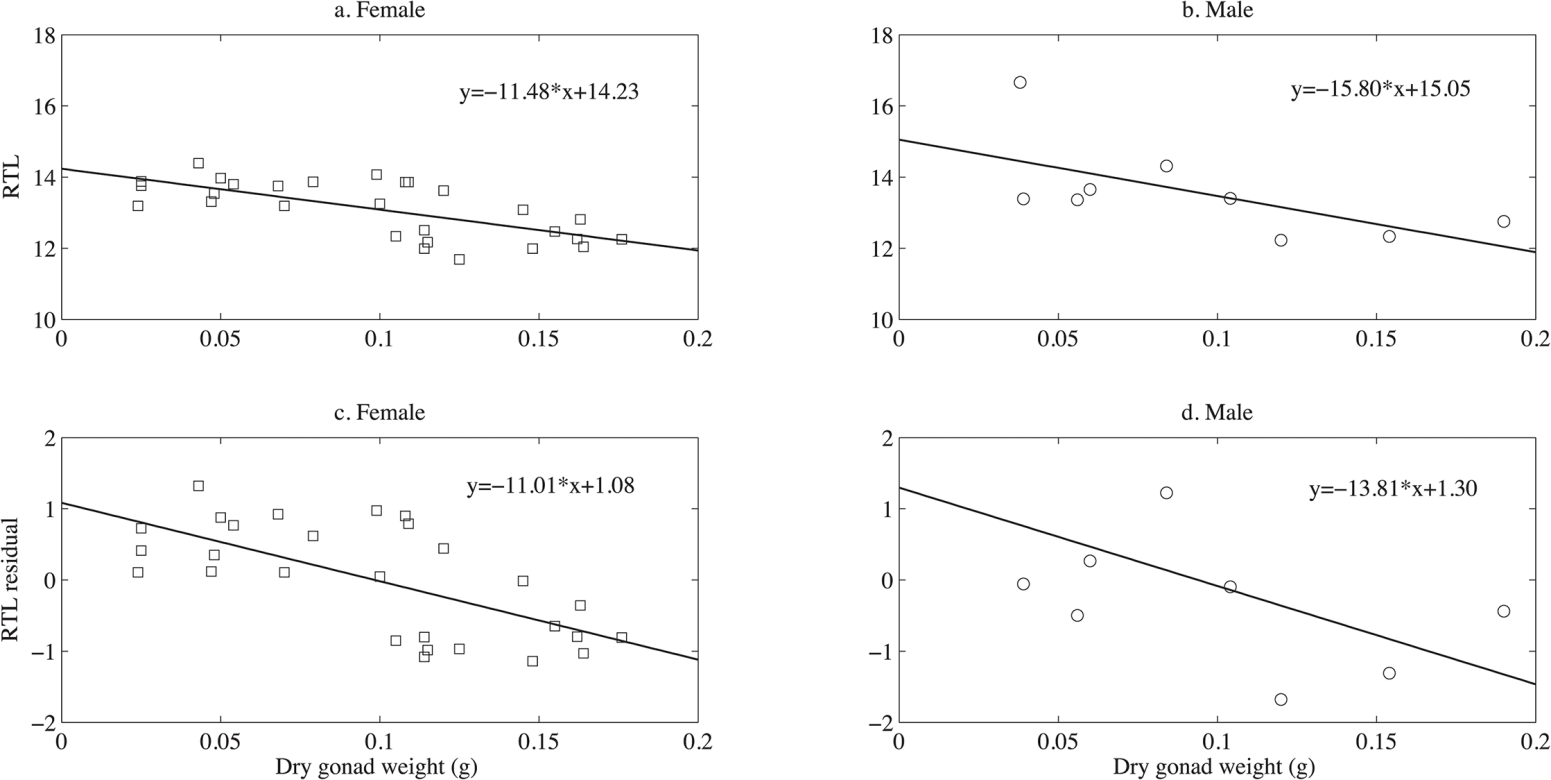
The relationship between RTL and dry gonad weight in muscle samples. Females are shown in a and c and males are shown in b and d. The RTL vs. dry gonad weight is shown in a and b and the RTL residuals from length effect vs. dry gonad weight are shown in c and d. The lines are fitted linear regressions.

In contrast, there was clear evidence of a trade-off between the telomere length and investment in reproduction on day 200 (Figs 4 and 5, see Table 4 for standard error and R^2^). To factor out the length effect on gonad size, length residuals were also tested against gonad mass in the linear regression. RTL in muscle tissue declined with total gonad mass in females and males. RTL in muscle tissue declined with total gonad mass in both females (RTL: slope = -11.48, p<0.001, Length residuals: slope = -11.01, p<0.001) and males (RTL: slope = -15.80, p = 0.07, length residuals: slope = -13.81, p = 0.1). The RTL in the brain tissue shows the same general trend (♀: RTL: slope = -6.54, p = 0.07, Length residual: slope = -8.67, p = 0.001, p = ♂: RTL: slope = -11.42, p = 0.21, Length residual: slope = -5.15, p = 0.46).

**Table 4:** The regression statistics of RTL and RTL length residuals on dry gonad mass separated by tissue and gender.

		RTL Muscle	Residual Muscle	RTL Brain	Residual Brain
Female	slope	**-11.48**	**-11.01**	-6.54	**-8.67**
	SE	2.42	2.44	4.10	2.33
	R^2^	0.46	0.44	0.16	0.42
	p	<0.001	<0.001	0.07	0.001
Male	slope	-15.8	-13.81	-11.42	-5.15
	SE	7.39	7.58	8.22	6.59
	R^2^	0.39	0.32	0.24	0.09
	p	0.07	0.10	0.21	0.46

The significant values are in bold and SE stands for standard error. The significance test for p<0.001 means that p is within the interval [0, 0.001].

**Fig 6:**
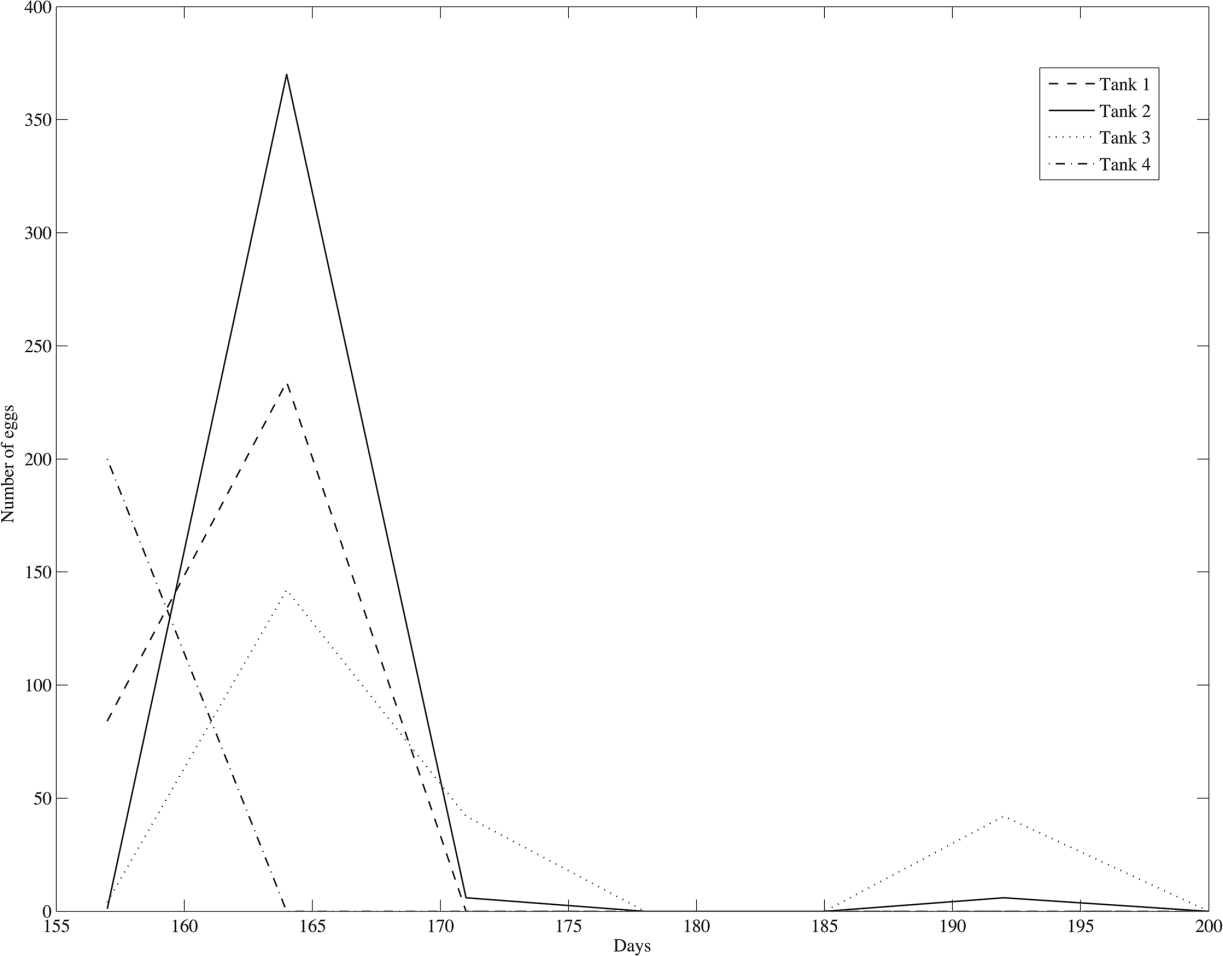
The egg production over time in the four replicates from day 150 to day 200.

The apparent trade-off between reproductive investment and telomere length was also present in total egg production (Fig 4). The average telomere length in the muscle tissue decreased significantly with the average fecundity (slope = -0.0090+/-0.02, R^2^ = 0.93, p<0.001). Similarly RTL in brain tissue decreased with fecundity, though this result was not significant (slope = -0.0040+/-0.003, R^2^ = 0.61, p = 0.40). The weekly egg counts in each tank are shown in Fig 6. Eggs were produced in large quantity from day 150 to 178 days and then at a very low rate until the end of the experiment.

**Fig 7:**
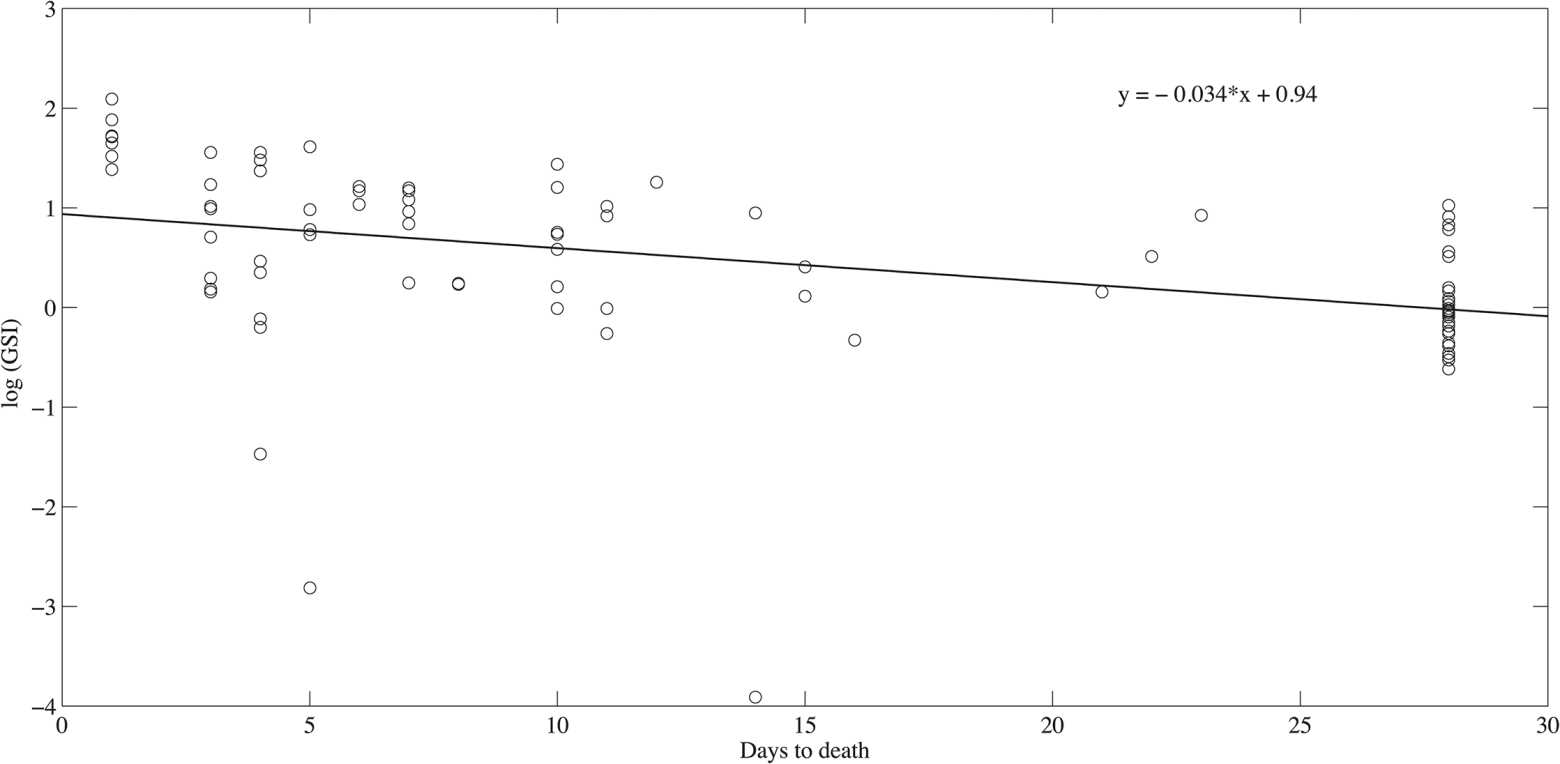
The relationship between the log(GSI) and days to death. The fitted line is the linear regression line.

In the independent study of reproduction vs. lifespan, we found a significant negative relation between time to death and ln[GSI] (slope = -0.034+/-0.01, R^2^ = 0.30, P<0.001) (Fig 7). Hence individuals with larger GSI died sooner than those with lower reproductive investment.

## Discussion

With few exceptions, telomeres have been found to shorten with age in a wide variety of taxa ranging from reptiles to birds and then to humans [7, 34–36]. Telomere length has been proposed as a surrogate for age [7, 35] or as a measure remaining life span [6]. This is particularly valuable for species that are hard to age or cannot be aged in a non-lethal manner.

However, it appears that fishes do not conform to this general pattern. In keeping with results for freshwater fishes [12–17] and marine species [22–24] there is no steady decline in telomere length with age in Atlantic silversides. Our results are not consistent with findings in closely related species either. According to recent phylogenetic study of Atherinomorpha which includes medakas, flyingfishes, killifishes, silversides, and their relatives, these fishes belong to a monophyletic group [37]. In Japanese medaka (*Oryzias latipes*), telomeres shorten during aging despite the considerable amount of telomerase activity and the telomere length is inversely correlated with the increase in body length. Similar to silversides, the platyfish (*Xiphophorus*) do show homogenous telomere lengths among tissues, which indicate a good model for telomere shortening research, but the age-dependency has not been investigated yet [18]. The actual mechanisms that regulate telomere length in silversides are as yet unknown. However, other fishes show significant telomerase activity throughout life including zebra fish [12, 13] and rainbow trout (*Oncorhynchus mykiss*)[38]. Variations telomerase activity throughout life undoubtedly contributes to the variation in rates of shortening observed.

Compared with the vertebrate taxa that do show telomere shortening though, these fish species are distinct in having ‘indeterminate’ or asymptotic growth. As has been hypothesized for senescence generally [25, 39], we suspect that telomere shortening should be delayed if fecundity increases sufficiently with age. If we consider telomere length as an indicator of remaining life expectancy, our results provide indirect support for the disposable soma theory of ageing [40] which postulates that senescence evolves as the optimal solution to an energetic trade-off between somatic maintenance and other life history characters. Corroborating the idea that reproductive investment is costly [see e.g. 41–45] and consistent with observations in other fishes [46–48], we found a significant decline in residual lifespan with increased investment in gonad. We note, however, that these fish were collected at the end of the breeding season, and relative gonad mass may therefore not be an ideal index of reproductive investment. Thus, some caution is needed in interpreting this result.

Nevertheless, our finding that increased fecundity is associated with decreased telomere length, supports the view that the rates of telomere shortening are adaptive and may trade-off against other energetically costly components of fitness [26, 49–51]. In light of the great diversity of life history strategies fishes display, we suspect that such trade-offs may contribute to the variety telomere shortening patterns found.

## Supporting Information

S1 Complete Experimental Protocol (DOCX)

S1 Dataset (XLS)

S1 Fig Confirmation of PCR amplification specificities of Mc1r gene. a. Gel electrophoresis of the PCR products was performed on 1% agarose gel. b. There was a single peak and the melting temperature was 87°C. c. The standard curve constructed by two 5-fold serial dilutions. Each dilution was run by qPCR in duplicate.
(EPS)

S2 Fig Confirmation of PCR amplification specificities of the telomere reaction. a. Gel electrophoresis of the PCR products was performed on 1% agarose gel. Two fish species (*G*. *morhua* and *M*. *menidia*) where tested and both of them showed a smear consistent with telomeres. b. The standard curve constructed by a 10-fold serial dilution using *M*. *menidia* sample. Each dilution was run by qPCR in duplicate.
(EPS)
