# Supplementary material for: Does Reproductive Investment Decrease Telomere Length in Menidia menidia?
Source: PLoS One. 2015 May 4;10(5):e0125674. doi: 10.1371/journal.pone.0125674 (PMC4418813; doi:10.1371/journal.pone.0125674)
Supplement: S1 Complete Experimental Protocol — (DOCX) [file pone.0125674.s001.docx]

**Supporting information 1:** *Complete experimental protocol*

**1.a DNA template**

Fresh tissue (entire brain and 0.2g muscle) samples were dissected out immediately after sampling and stored at -80°C until DNA extraction. The DNA was extracted using a Wizard® Genomic DNA Purification Kit (Promega) following the instructions for animal tissue in the manual. The quality and concentration of the extractions were then checked using Nanodrop 1000 (Thermo Scientific).

**1.b Primer design**

Although we did not determine copy number for Mc1r in *Menidia*, it is known to be a single copy gene in many teleost species [33]. We cloned (QIAGEN Plasmid Kit) and sequenced the plasmids at the DNA Sequencing Facility (Health Science Center) at Stony Brook University. Candidate primer sets were designed in Primer 3 (Whitehead Institute for Biomedical Research) and ordered from IDT (Integrated DNA Technologies). The primer set for Mc1r (150bp) in this study was forward: GTCCTCCCTCTCGTTCCTGT and reverse: AAGAGGATGCTGGACGTGAT. The Genbank accession number to the complete sequence was KP794198. Electrophoresis was run to confirm that there are no non-specific amplicons in regular PCR product (S1 Fig.1.a). The melting curve of the qPCR result also confirmed single amplicon in the reactions (S1 Fig.1.c).

The telomere primer sets used were tel1: GGTTTTTGAGGGTGAGGGTGAGGGTGAGGGTGAGGGT and tel2: TCCCGACTATCCCTATCCCTATCCCTATCCCTATCCCTA as described in Cawthon (2002). We confirmed the product by running gel electrophoresis on the PCR product (S1 Fig.1.a).

**1.c qPCR**

All qPCRs were performed on Mastercycler® ep realplex 2 S system (Eppendorf), a thermal cycler equipped to excite and read emissions from fluorescent molecules during each cycle of the PCR. We used 25μl systems suggested by SYBR GreenER™ qPCR SuperMix Universal Manual (Invitrogen), which includes 2 μl (1nmol/μl) of Primer, 2 μl of sample, 12.52 μl SYBR mix and 8.52 μl water. The DNA was diluted to 0.2 ng/μl to stay within the linear range of the standard curve for each amplification (see 1.d). Telomere (T) PCRs and single copy gene (S) PCRs were performed in separate transparent 96-well plates (Fisher Scientific). Repeated measures of the T/S ratio in the same DNA sample gave the lowest variability when the sample well position for T on the first plate matched the well position for S on the second plate. Two ‘master-mixes’ (SYBR GreenER™ qPCR SuperMix Universal, Invitrogen) were created for S and T respectively. We used clear plates with adhesive sealing method. Two replicates were run for each sample and two no-template controls were (replace sample with PCR water) also included on each plate.

The thermal cycling profile for both amplicons began with a 95°C incubation for 10 min to activate the AmpliTaq Gold DNA polymerase. For telomere PCR, there followed 40 cycles of 95°C for 15 s and 54°C for 2 min. Similarly for S, optimal conditions were 40 cycles of 95°C for 15 s and 56°C for 30s.

In order to confirm that there are no non-specific PCR products, a melting curve was constructed for the Mc1r product (S1 Fig.1.b). The melting process of double-stranded DNA causes a sharp reduction in the fluorescence signal (l) around the melting temperature (T_m_) of the PCR product, resulting in a clear peak in –dl/dT. Thus, a single peak indicates one specific product.

**1.d Standard curve construction and validation of the** $\boldsymbol{\Delta\Delta}$**C_t_ method**

Eppendorf’s Realplex 2.0 generated the standard curve for each gene (S1 Fig.1.c and S1 Fig.2.b). Serial dilution method was used. Specifically, C_t_ values were in each dilution were plotted against their corresponding DNA concentration. The standard curve was generated by a linear regression of the plotted points. The amplification efficiency E was then calculated as ${10}^{-1/slope}-1$ (Rasmussen 2001).

For the $\Delta\Delta$C_t_ method to be valid, the amplification efficiencies of the target and reference gene must be approximately equal. From the generated standard curves, the Mc1r assay had a slope of -3.10, Y-intercept of 24.32, R^2^ of 0.97 and efficiency of 110%. Telomere assay had a slope of -3.11, Y-intercept of 10.44, R^2^ is 0.99 with efficiency of 110%. The slopes and efficiencies of the two assays were similar enough for the $\Delta\Delta$C_t_ method to be valid (Livak and Schmittgen 2001).

Each plate carried a duplicate of the same three standard samples. The measured C_t_ was then adjusted up or down by the same percentage of the average of these standard sample C_t_. The inter-plate Ct variation was 1.8% for the Mc1r assay and 3.9% for the telomere assay. The within plate C_t_ variation was 1.0% for the Mc1r assay and 1.5% for the telomere assay. The coefficient of variation in $\Delta C_{t}$ (T/S) wass 6.7% between plates and 4.4% within plate. No template controls are also included in each plate. The measured C_t_ for these negative controls across all the plates was 33.7+/-1.33 for the Mc1r and 21.3+/-0.75 for the telomere assays.

**1.e Relative quantification of telomere length via** $\boldsymbol{\Delta\Delta}$**C_t_ method**

The T/S ratio was determined based on the C_t_ value for each gene and is approximately $2^{-\Delta C_{t}}$ where $\Delta C_{t}$ equals to the difference between telomere and the reference gene (Cawthon 2002). The relative T/S ratio of one sample to another sample is then $2^{-(\Delta C_{t1}-\Delta C_{t2})}=2^{-\Delta\Delta C_{t}}$.

**Figures**

**S1 Fig.1 Confirmation of PCR amplification specificities of Mc1r gene.**

a. Gel electrophoresis of the PCR products was performed on 1% agarose gel. b. There was a single peak and the melting temperature was 87°C. c. The standard curve constructed by two 5-fold serial dilutions. Each dilution was run by qPCR in duplicate.

**S1 Fig.2 Confirmation of PCR amplification specificities of the telomere sequences.**

a. Gel electrophoresis of the PCR products was performed on 1% agarose gel. Two fish species (*G. morhua* and *M. menidia*) where tested and both of them showed a smear consistent with telomeres. b. The standard curve constructed by a 10-fold serial dilution using *M. menidia* sample. Each dilution was run by qPCR in duplicate.

**Supporting information 2:** *Dataset*

**References**

Livak KJ, Schmittgen TD. Analysis of relative gene expression data using real time quantitative PCR and the $2^{-\Delta\Delta Ct}$ method. Methods. 2011;25: 402-408.

Rasmussen R. In: Meuer S, Wittwer C, Nakagawara K (Eds.), Quantification on the LightCycler. 2011. Springer-Verlag, Berlin, Germany, p21-34.
